# *Tle6* deficiency in male mice led to abnormal sperm morphology and reduced sperm motility

**DOI:** 10.3389/fcell.2024.1481659

**Published:** 2024-10-24

**Authors:** Kousuke Kazama, Yuki Miyagoshi, Hirofumi Nishizono

**Affiliations:** ^1^ Research Support Center, Medical Research Institute, Kanazawa Medical University, Uchinada, Japan; ^2^ Faculty of Life and Environmental Sciences, University of Yamanashi, Kofu, Japan

**Keywords:** transducin-like enhancer of split 6, subcortical maternal complex, infertility, male germ cell, sperm

## Abstract

Infertility affects over 15% of the global population, and genetic mutations are a substantial cause of infertility. Recent studies have focused on the subcortical maternal complex and its role in early embryonic development. TLE6, a core protein in the subcortical maternal complex, is crucial for female fertility; however, its role in male germ cells remains poorly understood. In this study, we generated a novel *Tle6* knockout mouse model using CRISPR-Cas9 to examine the impact of *Tle6* mutations on male fertility. *Tle6* knockout males exhibited a reduced total sperm count compared to wild-type mice, with a marked decrease in highly motile sperm. Histological observation of *Tle6* ^+/−^ mouse testes showed no apparent structural changes, though impaired sperm maturation was observed. Immunofluorescence staining showed that TLE6 localizes to the midpiece of sperm. It was also confirmed that the expression of *Tle6* is reduced in *Tle6* ^+/−^ male mice. In addition, *Tle6* ^+/−^ mice exhibited a significant increase in serum testosterone levels compared to wild-type mice. Changes in the expression of genes related to sperm function were also observed in the testes of *Tle6* knockout mice. These findings suggest that TLE6 is involved in sperm production and function, and that mutations in TLE6 may impair the production of functional sperm in humans, potentially leading to infertility.

## 1 Introduction

Currently, it is estimated that infertility impacts more than 15% of the global population, thereby representing a considerable global challenge (WHO, 2023; Vander Borght and Wyns, 2018). To date, numerous studies on infertility have revealed that infertility can be partly attributed to genetic mutations (Jiao et al., 2021). However, the mechanisms through which individual genetic variants can cause infertility are yet to be comprehensively elucidated. Although recent studies have reported early embryonic infertility due to impaired oocyte maturation, fertilization, and early embryo development (Jiao et al., 2021), several of these mechanisms remain unknown.

Among the cases of early embryonic infertility, infertility resulting from mutations in subcortical maternal complex (SCMC)-related genes has recently garnered considerable attention (Bebbere et al., 2014). SCMC reportedly plays a role in embryo development and cleavage (Yu et al., 2014). SCMC is essential for maintaining the unique structure of cytoplasmic lattices (CPL) within the oocyte cytoplasm, which is crucial for embryogenesis (Jentoft et al., 2023). CPLs accumulate proteins necessary for embryogenesis, such as tubulin, as well as proteins that regulate post-fertilization reprogramming, and temporarily inhibit the access of these accumulated proteins to the nucleus (Jentoft et al., 2023), thereby regulating proper embryonic development. Furthermore, proteins involved in genomic imprinting and embryonic genome activation accumulate within CPLs (Giaccari et al., 2024), underscoring the importance of SCMC-related genes in embryo development and genomic imprinting.

SCMC is composed of multiple protein groups, including the transducin-like enhancer of split 6 (TLE6), PADI6, and OOEP (Chi et al., 2024), and its structure is widely conserved among mammals (Bebbere et al., 2014; Bebbere et al., 2021). TLE6 is the most crucial SCMC member. In the absence of TLE6, the structural integrity of SCMC and CPL is compromised (Chi et al., 2024). Consequently, in TLE6-deficient mice, cell division gradually fails after the two-cell stage, resulting in embryo fragmentation and complete embryonic lethality at the morula stage (Yu et al., 2014). In humans, pathogenic mutations in TLE6 have been reported to cause early embryonic lethality, as demonstrated by investigations conducted in multiple countries (Alazami et al., 2015; Lin et al., 2020; Akbari et al., 2022). This accumulated evidence strongly suggests that TLE6 is an essential protein in female germ cells.

Although the function of TLE6 in female germ cells and its role in infertility have been reported, little is known regarding the importance of TLE6 in male germ cells. Feng et al. reported the cell cycle and proliferation rate following the knockout (KO) of the Tle6 gene in mouse spermatogonia using the CRISPR-Cas9 system. The authors found that the loss of TLE6 affected spermatogonia cell cycle progression, causing a slight delay in the cell growth rate (Feng et al., 2020). However, this study was limited to investigating the effects of *Tle6* KO on mouse spermatogonia, and further research is required to explore its effects on other cell types *in vivo*. In addition, human case reports have focused only on females within families, and reproductive function has only been assessed in males with *Tle6* mutations (Akbari et al., 2022). Investigating whether male carriers of Tle6 mutations can transmit these mutations to the next generation is important.

In this study, we examined the pathological effects of knocking down *Tle6*, which is known to affect female germ cells only, in male mice to better understand the effects of *Tle6* mutations and deletions on reproduction. Our results revealed that *Tle6* KO mice have a low contribution rate to germ cells, making it difficult to pass these mutations and deletions to the next generation. Based on findings, males with TLE6 mutations are less likely to pass these mutations to their offspring.

## 2 Materials and equipment

### 2.1 Animals

C57BL/6N male mice (8 weeks old) and female mice (3 weeks old), as well as ICR female mice (8 weeks old) used for embryo transfer, were purchased from Japan SLC, Inc. (Hamamatsu, Japan). Vasectomized male ICR mice were bred and maintained at our facility. All animals were pathogen-free and housed in individually ventilated cages (TECNIPLAST S.p.A., Buguggiate, Italy). Animals were maintained under a 12:12-h light-dark cycle at 22°C ± 2°C and 40%–60% relative humidity, with free access to food (CE2, CLEA Japan, Inc., Tokyo, Japan) and water. All animal protocols were reviewed and approved by the Institutional Animal Care and Use Committee of Kanazawa Medical University. All experiments were conducted in accordance with the Animal Experimentation Guidelines of the Kanazawa Medical University.

### 2.2 Preparation of mouse embryos

Embryos were prepared by *in vitro* fertilization (IVF) based on our previous reports (Nishizono et al., 2020). HTF medium (ARK Resource Co., Ltd., Kumamoto, Japan) was used for sperm preculture and IVF, and KSOM medium (ARK Resource Co., Ltd.) was used for embryo washing. IVF was performed using fresh or frozen sperm. IVF using frozen sperm was performed as reported previously (Ishizuka et al., 2013) with HTF containing 1 mM reduced l-glutathione (G6013; Sigma-Aldrich Co. LLC, St. Louis, MO, United States) for pre-culturing thawed sperm. For freezing and thawing of sperm, a commercial kit (FERTIUP, Kyudo Co., Ltd., Tosu, Japan) was used according to the manufacturer’s protocol. After removing extra sperm and cumulus cells, embryos were cultured in KSOM at 37°C.

### 2.3 Generation of *Tle6* KO mice using CRISPR-Cas9 system

To generate *Tle6* KO mice, crRNAs were designed using CRISPR-direct (https://crispr.dbcls.jp/) (Naito et al., 2015) and Benchling (Benchling, San Francisco, CA, United States, https://www.benchling.com/). Information regarding the primers used for PCR genotyping is provided in Supplementary Table S1. TracrRNA, crRNA, and the Cas9 protein were purchased from Integrated DNA Technologies, Inc. (IA, United States). To generate *Tle6* KO mice, we performed embryo genome editing using an electroporator (Nepa Gene Co., Ltd., Chiba, Japan) according to the method described in our previous report (Nishizono et al., 2020). After electroporation, embryos were transferred into recipient mice at the 2-cell stage.

### 2.4 Genotyping

DNA was extracted from the ear tissue using the HotSHOT method (Schapira et al., 2017). Specifically, ear tissues from ≥ 2-week-old mice were added to 180 µL of 50 mM NaOH (37439-95; Nacalai Tesque, Inc., Kyoto, Japan) and heated at 95°C for 10 min. After alkaline treatment, 20 µL of 1 M Tris HCl (pH 8.0; 06938-44; Nacalai Tesque, Inc.) was added to the sample. Extracted genomic DNA was used as a template for PCR genotyping. PCR was performed using KOD FX NEO (TOYOBO Co., Ltd., Osaka, Japan) according to the manufacturer’s instructions. Information regarding the primers used for PCR genotyping is provided in Supplementary Table S1. After purifying the extracted genomic DNA using NucleoSpin^®^ Gel and PCR Clean-up (Takara Bio Inc., Shiga, Japan), Sanger sequencing was performed using the services of Eurofin Genomics (Eurofins Genomics K.K., Tokyo, Japan). Sequence data were aligned with reference sequences using benchmarking to confirm the deletion of *Tle6*.

### 2.5 Histological examination of mouse testes with hematoxylin-eosin (H&E) staining

H&E-stained sections of testicular tissues were prepared using the histology services at Kanazawa Medical University following standard protocols. Briefly, the testes were collected from wild-type and *Tle6* ^+/−^ male mice and fixed overnight in 4% paraformaldehyde-phosphate buffer (PFA: 09154-14; Nacalai Tesque, Inc.). The specimens were dehydrated in graded alcohol solutions and immersed in xylene for dialysis. After immersion in paraffin for 12 h, the specimens were embedded in paraffin blocks, and sections of 3–4 µm thickness were prepared using a rotary microtome. Sections were then deparaffinized with xylene, rehydrated, dexylenized in graded alcohol solutions, and stained with H&E. The prepared H&E-stained slides were observed using a BZ-X700 microscope (Keyence, Osaka, Japan).

### 2.6 Measurement of testosterone levels

Blood was collected from mice via cardiac puncture and allowed to stand in the dark at room temperature for 15 min. The samples were then centrifuged at 3,000 rpm for 10 min, and the supernatant was collected. Serum testosterone levels were measured using a chemiluminescence immunoassay method, which was outsourced to the Japan Institute for the Control of Aging, Nikken SEIL Co., Ltd. (Shizuoka, Japan). Testosterone levels were measured using pooled serum samples from three wild-type and three *Tle6* ^+/−^ male mice, respectively.

### 2.7 Sperm count and motility analysis

Sperm were collected from the cauda epididymis of male mice and analyzed for number and motility using the Makler^®^ Counting Chamber (Sefi-Medical Instruments Ltd., Haifa, Israel). The sperm count and motility were measured according to the manufacturer’s instructions.

### 2.8 Quantitative PCR (qPCR) analysis of spermatozoa in mouse cauda epididymis

For the analysis of *Tle6* levels in sperm DNA, sperm were collected from the cauda epididymis of mice into approximately 30 μL of HTF medium. From this sample, sperm were randomly collected from three different locations. DNA was extracted using NucleoSpin DNA Rapid Lysis (Takara Bio, Inc.). Genomic DNA concentration was measured using a NanoDrop Lite Plus (Thermo Fisher Scientific, Inc., Waltham, MA, United States) and adjusted such that the DNA amounts were equal across all groups.

For the measurement of gene expression levels in the testis, the tissue was homogenized using TaKaRa BioMasher Standard (9791A; Takara Bio Inc.). Total RNA was then extracted and reverse-transcribed into cDNA using SYBR™ Green Fast Advanced Cells-to-CT™ Kit (A35379; Thermo Fisher Scientific Inc.). The cDNA was then used for qPCR to compare gene expression levels. The cDNA was obtained from three WT mice and three *Tle6* ^+/−^ mice and was divided into three technical replicates for each group.

The samples were then subjected to qPCR using the THUNDERBIRD™ Next SYBR^®^ qPCR Mix (TOYOBO Co., Ltd.) or SYBR™ Green Fast Advanced Cells-to-CT™ Kit according to the manufacturer’s protocols using the CronoSTAR 96 Real-Time PCR System (Takara Bio Inc.). The primers used for qPCR were designed using Primer3Plus (https://www.primer3plus.com/) and Blast (Supplementary Table S1).

### 2.9 Immunofluorescence staining

Immunofluorescence staining of sperm was performed with slight modifications following the method described by Yu et al. (2014). Details of the antibodies and DAPI are provided in Supplementary Table S2. In brief, frozen sperm were thawed at 37°C for 10 min and then fixed in 4% PFA with 0.2% Triton-X 100 (35501-02; Nacalai Tesque, Inc.). After blocking with 3% BSA in PBS containing 0.5% Triton X-100 at room temperature (RT) for 1 h, the samples were incubated with mouse anti-TLE6 antibody (1:500, SC-515065; Santa Cruz Biotechnology, TX, United States) at 4°C overnight. The samples were incubated with goat anti-Mouse IgG (1: 500, ab150113; Abcam, Cambridge, United Kingdom) at RT for 1 h. Finally, the samples were treated with DAPI (1 ng/mL, 19178-91; Nacalai Tesque, Inc.), and sperm morphology was observed using a fluorescence microscope (BZ-X 700) with glass-bottom dishes.

### 2.10 Observation of sperm morphology

After thawing frozen sperm at 37°C for 10 min, sperm were fixed in a tube containing 4% PFA at room temperature for 20 min. The samples were then centrifuged at 3,000 rpm for 5 min, followed by three washes with 0.1% Poly vinyl alcohol (341584; Sigma-Aldrich Co. LLC) dissolved in PBS. After centrifugation, the sperm were stained with DAPI (1 ng/mL) and observed for morphology using a confocal fluorescence microscope (LSM701; Zeiss, Jena, Germany).

### 2.11 Statistical analysis

To test the genotype distribution of the offspring, a chi-square goodness-of-fit test was performed against the standard genotype distribution. We used the Student’s t-test for other data comparisons. The number of spermatozoa and motile sperm was reported as mean ± standard deviation (SD). The significance level was set at *p* < 0.05, and two-tailed tests were applied. All the analyses were performed using Python 3 and Microsoft Excel.

## 3 Results

### 3.1 Generation of *Tle6* KO mouse lines using the CRISPR-Cas9 system

**FIGURE 1:**
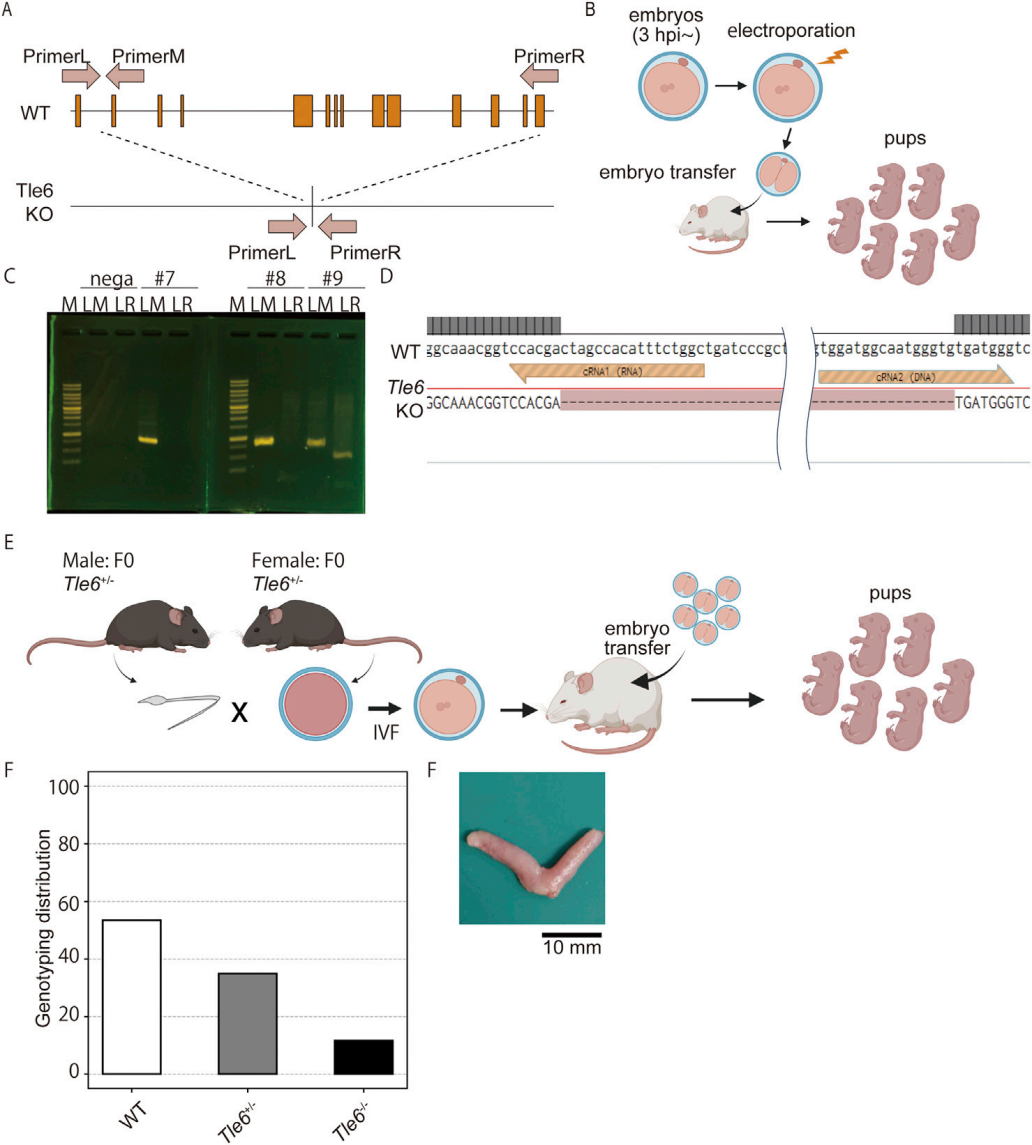
Generation of *Tle6* KO mouse lines using the CRISPR-Cas9 system. **(A)**, *Tle6* knockout (KO) design scheme. The upper row shows the wild-type (WT) *Tle6*. The lower row shows the KO design. The gRNAs were designed to delete the entire coding sequence (CDS) region, resulting in a KO size of 9.4 kb. **(B)**, The experimental scheme used to create *Tle6* KO embryos using performing genome editing of *in vitro* fertilized embryos. Embryos edited by electroporation were transferred to recipient mice at the two-cell stage, and pups were produced. **(C)**, Genotyping PCR of offspring derived from *Tle6* KO embryos. LM shows wild-type *Tle6*. LR shows *Tle6* after KO. **(D)**, The sequence of Founder mice with confirmed *Tle6* KO. The upper row (LM) shows a wild-type sequence. The lower row (LR) shows *Tle6* after KO. **(E)**, *In vitro* fertilization (IVF) scheme using male and female *Tle6^+/−^* KO founder mice. **(F)**, Genotype distribution of the offspring derived from embryos produced by IVF using *Tle6* ^+/−^ male and female mice (n = 3 replicates, 13 animals, 264 embryos). **(G)**, Representative photo of the uterus after cesarean section.

We generated *Tle6* KO mice to investigate the effects of *Tle6* deficiency in males. We performed genome editing of the embryos using the CRISPR-Cas9 system and electroporation to generate *Tle6* KO mice. The gRNA sequences were designed to knock out the entire *Tle6* coding sequence (CDS) (Figures 1A, B). Genotypes of mice born from genome-edited embryos were confirmed by genotyping PCR (Figure 1C), and deletion of the CDS in *Tle6* KO mice was determined by Sanger sequencing (Figure 1D). We transferred 978 two-cell embryos to pseudopregnant females to obtain 146 live pups. However, only a limited number of *Tle6* KO mice were obtained (WT: *Tle6* ^+/−^: *Tle6* ^−/−^ = 91.1%: 8.1%: 0%). No mosaicism was detected in the sequencing results.

To generate *Tle6* ^+/−^ male mice, we performed IVF and embryo transfer (ET) using *Tle6* ^+/−^ females and males (Figure 1E). We transferred 261 embryos into pseudo-pregnant females and obtained 23 WT, 15 *Tle6* ^+/−^, and five *Tle6* ^−/−^ mice (Figure 1F). According to Mendelian laws, the crossbreeding of heterozygotes should yield a ratio of one *Tle6* ^−/−^: two *Tle6* ^+/−^: one WT. However, the observed ratio was one *Tle6* ^−/−^: three *Tle6* ^+/−^: 4.6 WT. The genotype proportions differed significantly from the expected genotype proportions (χ^2^ = 19.0, *p* = 7.485 × 10^−5^). Upon examining whether fertilized eggs were arrested during embryonic development in the uteri of pseudo-pregnant females, we found no embryos with arrested development after implantation (Figure 1G). The substantially lower number of heterozygous embryos than anticipated was unexpected. Given that *Tle6* ^−/−^ mice were not obtained via IVF-ET, we used *Tle6* ^+/−^ male mice for subsequent phenotypic analysis.

### 3.2 *Tle6* KO is rarely transmitted to offspring through natural mating

**FIGURE 2:**
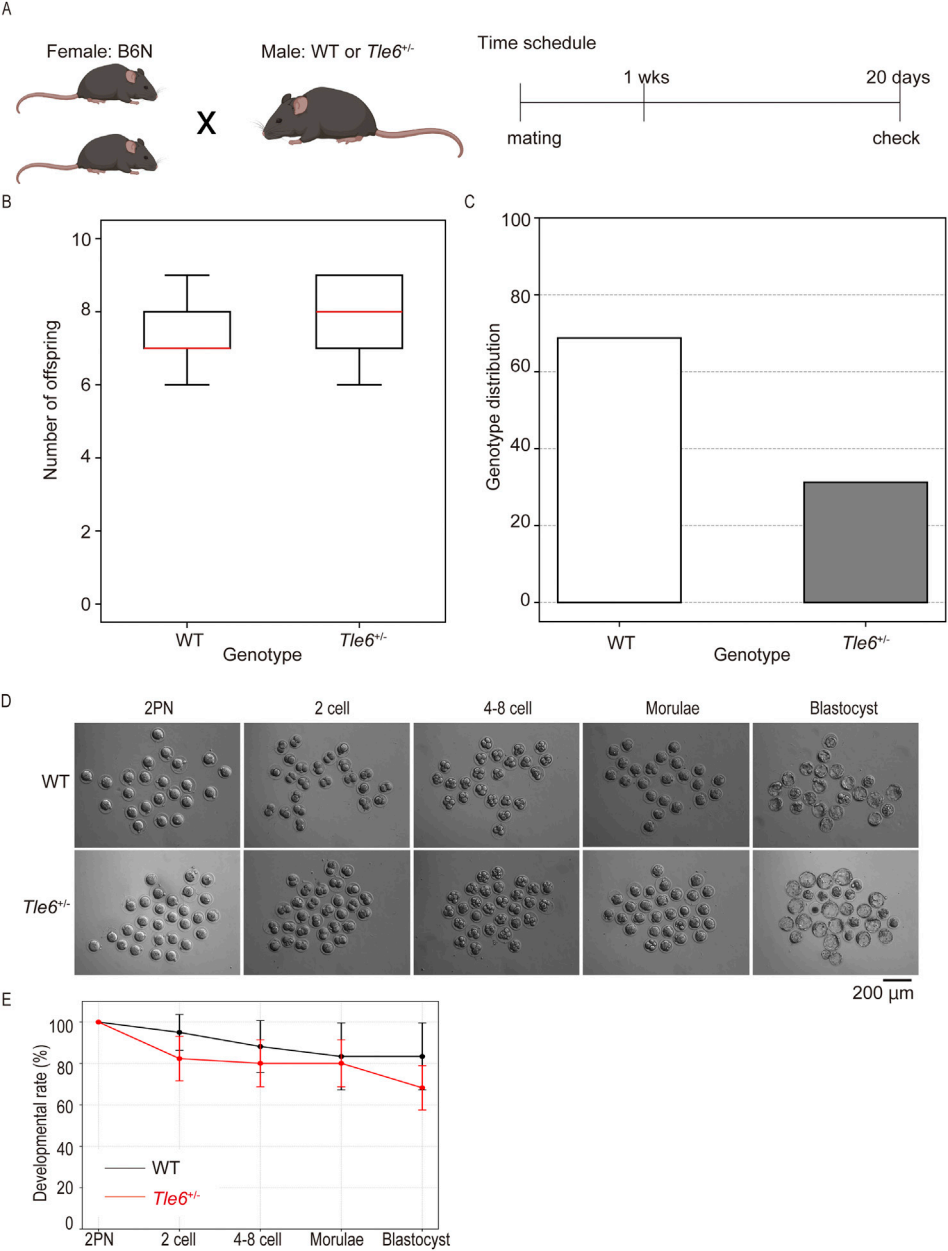
Natural mating using *Tle6^+/−^* male mice and wild-type (WT) female mice. **(A)**, Experimental scheme for natural mating using *Tle6* ^+/−^ Founder male mice and WT female mice. Two female mice were mated with one male mouse for 1 week. **(B)**, Number of offspring produced by natural mating using WT female mice and *Tle6* ^+/−^ or WT male mice (n = 4 replicates, 20 animals). **(C)**, Genotype distribution of offspring derived from *Tle6* ^+/−^ Founder male mice and WT female mice (WT *n* = 22, *Tle6^+/−^*: n = 10). **(D)**, Representative photos of embryos produced by *in vitro* fertilization using sperm from WT and *Tle6* ^+/−^ mice. **(E)**, Embryonic development rates of embryos produced by *in vitro* fertilization using sperm from WT and *Tle6* ^+/−^ mice (WT mice n = 4, embryos n = 112, *Tle6* ^+/−^ mice: n = 10, embryos n = 85).

First, to investigate whether *Tle6* deficiency affects mating behavior, we conducted breeding experiments using natural mating. Each *Tle6* ^+/−^ and WT male mice were mated with two C57BL/6N WT female mice for 1 week, and mating was confirmed 20 days later (Figure 2A). Herein, we found that the mating frequency of *Tle6* ^+/−^ male mice did not differ from that of WT mice (each at 62.5%), and the number of offspring was also comparable to that of WT mice (Figure 2B, WT: *Tle6* ^+/−^ = 7.40 ± 1.14: 7.80 ± 1.30, *p* = 0.6195). These results suggest that *Tle6* deficiency does not affect mating behavior or fertility.

The genotypes of F1 mice born through natural mating were confirmed by PCR and Sanger sequencing. Interestingly, most mice were WT, and very few *Tle6* ^+/−^ mice were obtained (Figure 2C). According to Mendelian inheritance, *Tle6* ^+/−^ and WT mice should be born in a 1:1 ratio when *Tle6* ^+/−^ and WT mice are bred. However, the results of this experiment differed significantly (χ^2^ = 4.5, *p* = 0.0339). This phenomenon is consistent with the results shown in Figure 1F.

It is known that *Tle6*-deficient oocytes are embryonic lethality, then we hypothesized that embryos derived from *Tle6* KO sperm might exhibit similar reduced developmental rate. To exam this hypothesis, we performed IVF using *Tle6* ^+/−^ male mice sperm and *in vitro* culture (Figure 2D). However, embryos derived from *Tle6* ^+/−^ male mice sperm showed similar developmental rate compared to that derived from WT male mice sperm (Figure 2E). These results indicated that *Tle6* ^+/−^ mice exhibited specific abnormalities in sperm rather than atypical mating behavior or fertility, hindering the transmission of the *Tle6* KO to the next generation.

### 3.3 *Tle6^+/−^* mice are able to produce spermatozoa; however many of them exhibit abnormal morphology and low motility

We hypothesized that the difficulty in transmitting genetic traits from *Tle6^+/−^* male mice could be due to reduced sperm count and motility. If the number and motility of *Tle6* KO sperm are decreased, sperm carrying *Tle6* KO genetic trait would likely be unable to swim to the oviduct ampulla in the female reproductive tract and fertilize the oocytes. Consequently, the likelihood of *Tle6* KO genetic trait being passed on to the next generation would be reduced. To test this hypothesis, we analyzed the testes and sperm of *Tle6* ^+/−^ male mice.

**FIGURE 3:**
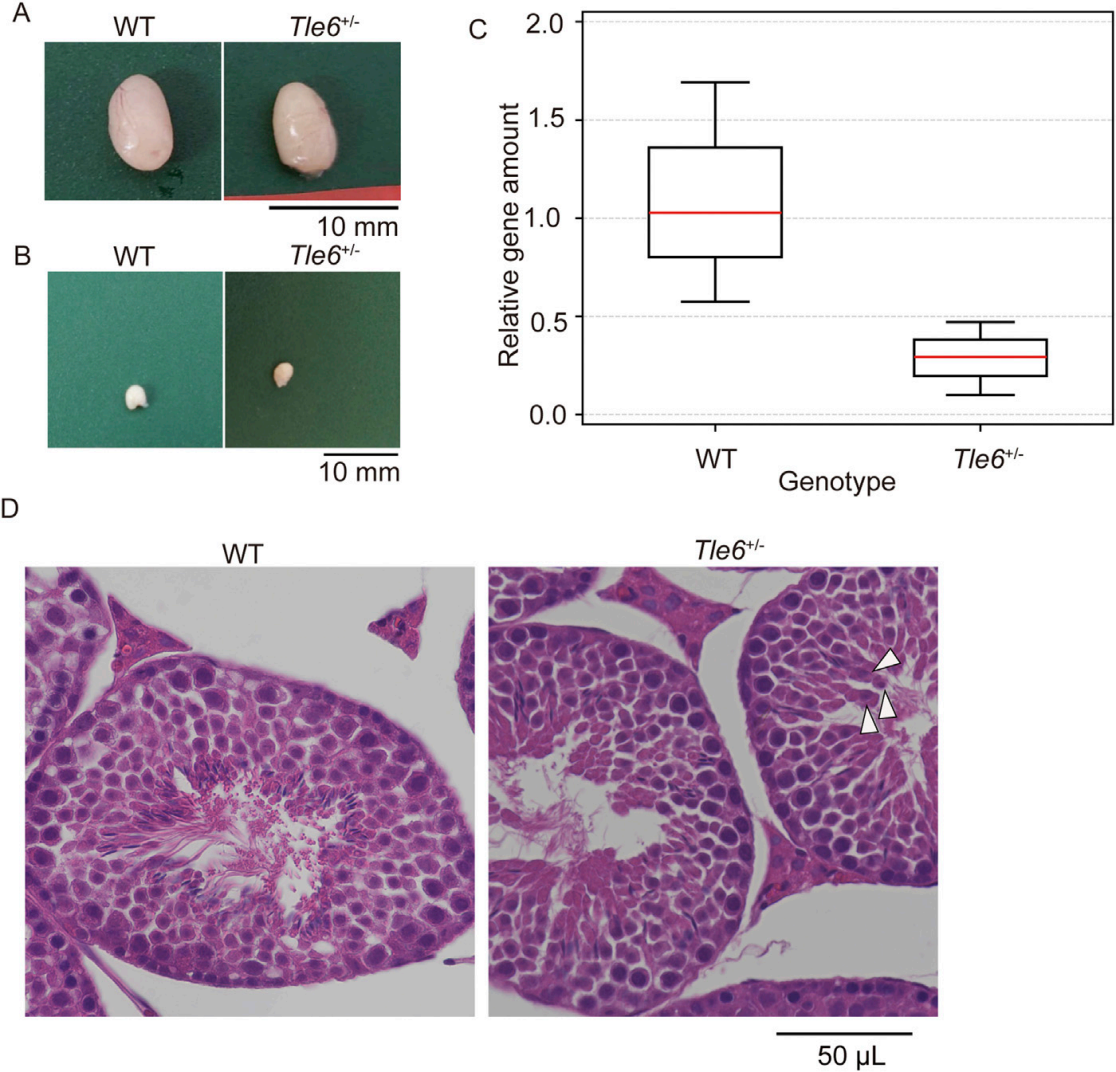
Analysis of sperm production in the male reproductive tissues of WT and *Tle6^+/−^* mice **(A)**, Testis of WT C57BL/6N mice (left) and *Tle6* ^+/−^ mice (right) **(B)**, Cauda epididymis of WT C57BL/6N mice (left) and *Tle6* ^+/−^ mice (right). **(C)**, qPCR of sperm from WT C57BL/6N mice and *Tle6* ^+/−^ mice (n = 3 replicates). **(D)**, Hematoxylin and eosin staining of testes of WT C57BL/6N mice (left) and *Tle6* ^+/−^ mice (right). Arrowhead indicates spermatids near the center of the seminiferous tubules.

First, we compared the testes of WT and *Tle6* ^+/−^ mice and found no morphological abnormalities (Figure 3A). Similarly, no morphological abnormalities were found in the cauda epididymis of *Tle6* ^+/−^ male mice (Figure 3B), and there was no significant difference in the size of the cauda epididymis between the two groups (WT: *Tle6* ^+/−^ = 2.98 ± 0.21 mm: 3.08 ± 0.10 mm, *p* = 0.426, Student’s t-test).

Next, we examined whether *Tle6* KO spermatozoa was suppressed in the testes of *Tle6* ^+/−^ male mice. Consequently, we performed qPCR using spermatozoa derived from WT and *Tle6* ^+/−^ male mice. For qPCR, spermatozoa were collected from three regions of the cauda epididymis from both WT and *Tle6* ^+/−^ mice. The analysis was conducted using a consistent amount of DNA extracted from sperm genomes. Since the primers for qPCR were designed at the *Tle6* knockout site, it was anticipated that the quantity of the *Tle6* gene in *Tle6* ^+/−^ mice would be half of that in WT mice. However, the *Tle6* gene quantity in spermatozoa derived from *Tle6* ^+/−^ mice was less than 30% of that in spermatozoa from WT mice (Figure 3C), with a relative level of 0.289 compared to sperm from WT mice (χ^2^ = 1.6637, *p* = 0.1971 × 10^−2^). No significant differences were observed between groups. These results indicate that *Tle6*-deficient sperm were present in the cauda epididymis of *Tle6* KO mice, and although there was no significant difference in the ratio, their number was higher than that of normal sperm, with an estimated 70% of sperm being deficient in *Tle6*. The reduced number of motile sperm in the semen of *Tle6* ^+/−^ mice could be attributed to the high proportion of *Tle6*-deficient sperm.

Finally, to investigate sperm abnormalities more thoroughly, we compared spermatogonia, testis structure, sperm count, motility, and sperm morphology. HE-stained sections of the testes were prepared using the testes of WT and *Tle6* ^+/−^ male mice. While no obvious differences in testis structure were observed, the interstitial tissue of *Tle6* ^+/−^ mice appeared a little shrunken. Suspecting that this shrinkage might be due to abnormalities in hormone levels, we analyzed serum testosterone levels in both WT and *Tle6* ^+/−^ male mice. The results showed that testosterone level in *Tle6* ^+/−^ mice were significantly higher than that in WT mice (WT: 651 ng/L, *Tle6* ^+/−^: 7,804 ng/L). Then, spermatids were occasionally detected near the center of the seminiferous tubules in *Tle6* ^+/−^ mice, and enucleated cytoplasmic remnants were also observed. These findings suggest that meiotic failure may occur in spermatids of *Tle6* ^+/−^ male mice (Figure 3D). The sperm count in *Tle6* ^+/−^ mice was significantly lower than in WT mice (Table 1, *p* = 0.012). Additionally, analysis of sperm motility in *Tle6* ^+/−^ mice showed a significantly reduced in number of motile sperm (Table 2).

**TABLE 1:** Sperm number of wild-type C57BL/6N mice and *Tle6* ^+/−^ mice.

	Mean ± SD (× 10^8^ sperm/mL)
WT	58.75 ± 7.54
*Tle6* ^+/−^	40.5 ± 7.19

Results are presented as mean ± standard deviation (SD). WT mice: (n = 2); *Tle6* ^+/−^ mice: (n = 2).

**TABLE 2:** Sperm motility of wild-type C57BL/6N mice and *Tle6* ^+/−^ mice.

× 10^8^ sperm/mL				
	1	2	3	4
WT	6.25 ± 4.57	10 ± 1.15	19.5 ± 7.33	41 ± 8.21
*Tle6* ^+/−^	6.75 ± 4.57	11.25 ± 1.71	10 ± 2.45	24.25 ± 5.12

Results are presented as mean ± standard deviation. WT mice (n = 2); *Tle6* ^+/−^ mice (n = 2). A score of 1 indicated sperm with no movement. A score of 2 indicates sperm with movement but no forward swimming. A score of 3 indicates sperm with movement and slow-forward swimming. A score of 4 indicates sperm with movement and vigorous forward.

**FIGURE 4:**
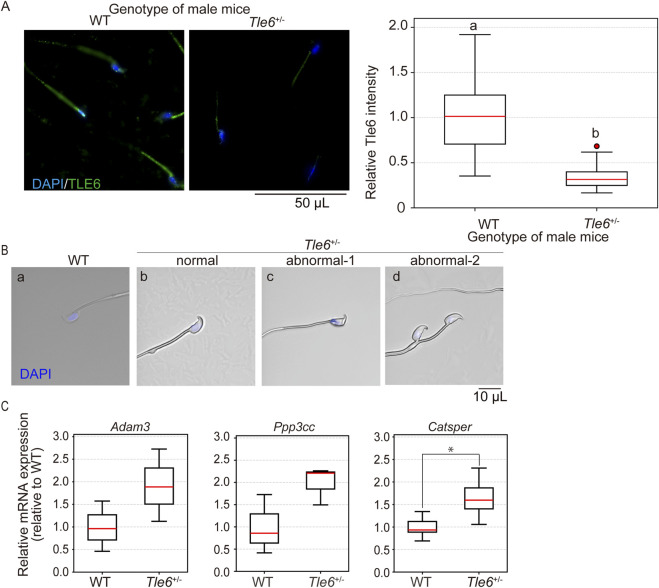
Analysis of testes and spermatozoa of *Tle6* KO heterozygous male mice **(A)**, Immunofluorescence staining of sperm from the cauda epididymis of WT and *Tle6* ^+/−^ mice: the left panel shows sperm from WT mice, and the right panel shows sperm from *Tle6* ^+/−^ mice. Blue indicates DAPI staining, and green indicates TLE6 staining. The lower graph shows the relative protein level of TLE6 in sperm, normalized to the average TLE6 protein level in sperm from WT mice (WT: sperm derived from wild-type male mice n = 41, *Tle6* ^+/−^: sperm derived from *Tle6* ^+/−^ male mice n = 32). **(B)**, Spermatozoa in cauda epididymis of WT mice (WT), normal sperm (normal), abnormal acrosome sperm (abnormal-1), and deformed sperm (abnormal-2) of *Tle6* ^+/−^ mice (three right panels). **(C)**, Quantification of the expression levels of fertilization-related genes in mouse testes analyzed by real-time PCR. The results for each gene were normalized to the average value from WT mice, with expression levels in both WT and *Tle6* ^+/−^ mice adjusted accordingly. The *X*-axis represents the genotype of the mice, and the *Y*-axis shows the relative expression levels compared to WT (n = 3 animals).

To assess *Tle6* protein levels in sperm, we performed immunofluorescence staining on sperm from WT and *Tle6* ^+/−^ mice. *TLE6* levels in *Tle6* ^+/−^ male mice were found to be less than half of those in WT mice (*p* = 5.013 × 10^−14^). Furthermore, *Tle6* was localized in the sperm midpiece. This localization partially overlapped with the mitochondrial sheath, suggesting that TLE6 might be involved in energy production in sperm (Figure 4A). Furthermore, we observed sperm morphology and found that while normal sperm were present, 57% sperm exhibited abnormal head shapes (crumpled appearance and displaced nuclei) and 7% double-headed sperm in *Tle6* ^+/−^ mice. These results suggest that *Tle6* deficiency leads to the production of abnormal sperm and reduces sperm count and motility.

The abnormalities in sperm from *Tle6* ^+/−^ mice suggest that genes related to fertilization may be affected. To investigate the downstream genes impacted by *Tle6* deletion, we assessed the expression levels of three key genes: *Adam3*, *Ppp3cc*, and *Catsper*, which are known to play roles in sperm motility, fertilization, and sperm morphology. The expression levels of these genes in testicular cells generally tended to be higher in *Tle6^+/−^* mice, with *Catsper* showing a significant increase (*Adam3*: *p* = 0.1806, *Ppp3cc*: *p* = 0.097, *Catsper*: *p* = 0.046).

## 4 Discussion

In this study, we generated novel *Tle6* KO mice to investigate the effects of TLE6 mutations and deletions on the male germline. The genotype distribution of the offspring did not follow Mendelian laws, indicating that the deletion of *Tle6* was unlikely to be transmitted to the next-generation (Figure 2C). Upon analyzing the genotype of sperm in the cauda epididymis of *Tle6* ^+/−^ mice, we detected higher numbers of *Tle6*-deficient sperm than WT sperm (Figure 3C). While deletion of *Tle6* did not have a major impact on the morphology of the testes or cauda epididymis (Figures 3A, B), a slight contraction of the testicular interstitium was observed (Figure 3D), along with a significant increase in testosterone levels. *Tle6* ^+/−^ mice had a lower sperm count than WT mice (Table 1). Notably, the number of motile sperm was reduced (Table 2). Moreover, many spermatozoa had abnormal morphology (Figure 4B). Additionally, gene expression related to fertilization, sperm motility, and sperm morphology in the testes of *Tle6* ^+/−^ mice showed an overall upward trend, with *Catsper* in particular showing a significant increase in expression (Figure 4C). The decrease in the number of healthy functional sperm, despite the high number of *Tle6*-deficient sperm in the cauda epididymis, suggests that *Tle6*-deficient sperm can process incorrect spermatogenesis and induce sperm dysfunction.

TLE6 is a component of the SCMC and plays an important role in the early embryonic development of oocytes and embryos (Jentoft et al., 2023). However, the function of TLE6 in male germ cells remains poorly clarified; therefore, we analyzed the role of TLE6 using newly generated *Tle6* KO mice. Our results indicate that, unlike previous reports (Yu et al., 2014), the genetic traits of *Tle6* ^+/−^ mice are rarely transmitted to the next generation (Figures 1F, 2C). This discrepancy is likely due to larger deletions in the genomic regions of our *Tle6* KO mice when compared with those performed in previous studies (Yu et al., 2014). In the current study, the generated *Tle6* KO mice had exons 2–14 deleted, whereas the mice used by Yu et al. had exons 1–2 replaced with a neomycin-resistant cassette. *Tle6* encodes the tryptophan-aspartic acid 40 repeat (WDR) domain in the CDS from exon 2 onward, and specific regions within this domain mediate interactions with other proteins forming the SCMC (Chi et al., 2024). Interestingly, the WDR domain is crucial for protein-protein interactions and is involved in several physiological activities, including the cell cycle (Schapira et al., 2017). It has also gained attention as a potential target for drug discovery. Therefore, this region likely contains elements critical for sperm function. It is highly likely that this crucial domain was not reliably deleted in previously reported mice. In future studies, we plan to analyze the protein function of the missing region in KO mice using a structural biology approach.

We also found that *Tle6* deficiency led to the production of abnormal sperm (Figure 4B) and a decrease in both the number and motility of sperm in the cauda epididymis (Tables 1, 2). However, the efficiency of natural mating in *Tle6* ^+/−^ mice was not reduced when compared with that in WT mice (Figure 2B). Considering these results, along with the comparison of sperm genotypes in the cauda epididymis shown in Figure 3C, it seems likely that even in the presence of substantial *Tle6*-deficient sperm with reduced motility and other phenotypes, the remaining motile WT sperm will fertilize the egg. Therefore, we speculate that the male genetic trait of *Tle6* deficiency is unlikely to be transmitted to the next generation. Feng et al. reported that the loss of *Tle6* in spermatogonia causes abnormalities in the cell cycle and proliferation rate of sperm cells. Our results showed a reduction in sperm count, which closely aligns with their findings. We also observed the presence of cells like spermatids in the center of the seminiferous tubules of *Tle6* ^+/−^ mice (Figure 3D). This observation suggests a failure in meiosis during spermatogenesis. To the best of our knowledge, this is the first report documenting a decrease in sperm in *Tle6* KO mice. Additionally, the abnormally high levels of testosterone may indicate dysfunction in the testes of *Tle6* ^+/−^ mice. While it is generally known that an increase in testosterone typically leads to a higher sperm count, no positive correlation was observed between changes in testosterone levels and sperm count in this study. Further research is needed to clarify this phenomenon.

Furthermore, we found that *Tle6* deficiency leads to the production of abnormal sperm (Figure 4B). These findings suggested that *Tle6* deficiency affects correct spermatogenesis. Although, we observed an upward trend in the expression of genes related to sperm motility and morphology in the testes of *Tle6* ^+/−^ mice, *Catsper* was the only gene that showed a significant increase (Figure 4C). These increase in gene expression is likely due to the knockout of *Tle6*, as TLE6 is known to function as a DNA corepressor.

It is important to note that this study focused on a specific region of *Tle6*-deficient mice (exon 2-14 knock out). While we observed a reduction in sperm count and motility, these symptoms have not been reported in other studies using different *Tle6* KO model mice. Moreover, the role of TLE6 in spermatogenesis may vary between humans and mice. Therefore, further research is necessary to clarify the mechanisms by which *Tle6* deficiency causes sperm abnormalities in *Tle6* KO mice and to explore its clinical relevance in humans.

In this study, we generated *Tle6* KO mice to determine the effects of TLE6 mutations on the male germline. *Tle6* ^+/−^ mice did not follow Mendelian inheritance and showed no morphological changes in the testes or spermatogonia. However, the production of sperm with abnormal morphology, as well as sperm count and motility, were significantly reduced, suggesting that *Tle6* deficiency results in incorrect spermatogenesis. While the effects of *Tle6* deficiency on males have rarely been reported, they may be related to potential male infertility. Nonetheless, these results are based on studies in mice and may not fully apply to humans.

## Data Availability

The original contributions presented in the study are included in the article/Supplementary Material, further inquiries can be directed to the corresponding author.
