# Supplementary material for: Tle6 deficiency in male mice led to abnormal sperm morphology and reduced sperm motility
Source: Front Cell Dev Biol. 2024 Oct 24;12:1481659. doi: 10.3389/fcell.2024.1481659 (PMC11540623; doi:10.3389/fcell.2024.1481659)
Supplement: Supplementary file 1 [file Table1.docx]

Supplementary Material

# Supplementary Tables

## Supplementary Table S1. The crRNA and primers used in the study.

| Tle6 crRNA1 | GCCAGAAAUGUGGCUAGUCGUGG |
| --- | --- |
| Tle6 crRNA2 | TGGATGGCAATGGGTGTGATGGG |
| Tle6 Genotyping primer L | GGGTGAGAGGGGTCAAATGG |
| Tle6 Genotyping primer M | GGGTGAGAGGGGTCAAATGG |
| Tle6 Genotyping primer R | GATCTGCACACCCCATGGAA |
| qPCR Tle6 primer L | TGGGTATTGTCCACACTCCAAC |
| qPCR Tle6 primer R | ATGTTTTGCCGTCTGAAGCC |
| qPCR Gapdh primer L | AAAAGAAGGTCGGGTGGAAGAG |
| qPCR Gapdh primer R | TCTTCTGGGTGGCAGTGATG |
| qPCR Catsper primer L | ATGCACAAATGACCGAGGAG |
| qPCR Catsper primer R | TCTGTTGATGCTGTTCTACCG |
| qPCR Adam3 primer L | GCTTTGCCATTGTTCTCTCAC |
| qPCR Adam3 primer R | GCTAAAAACCTTTATGCCTTGGG |
| qPCR Ppp3cc primer L | GCGCCCTCAGTTCTCCAC |
| qPCR Ppp3cc primer R | TCTTCCACCCGACCTTCTTTTAC |

## Supplementary Table S2. Staining reagents used in immunofluorescence staining.

| Product | Product number | Vendor | Dilution ratio | Dissolved buffer |
| --- | --- | --- | --- | --- |
| TLE6 Antibody (D-4) | sc-515065 | Santa Cruz Biotechnology | 1: 500 | Blocking buffer |
| Goat Anti-Mouse IgG H&L (Alexa Fluor® 488) | ab150113 | Abcam | 1: 500 | Blocking buffer |
| DAPI Solution | 19178-91 | Nacalai Tesque, Inc. | 1 ng/mL | PBS |
